# Integrative meta-analysis of differentially expressed genes in osteoarthritis using microarray technology

**DOI:** 10.3892/mmr.2015.3790

**Published:** 2015-05-15

**Authors:** XI WANG, YUJIE NING, XIONG GUO

**Affiliations:** School of Public Health, Xi'an Jiaotong University Health Science Center, Key Laboratory of Trace Elements and Endemic Diseases, National Health and Family Planning Commission, Xi'an, Shaanxi 710061, P.R. China

**Keywords:** osteoarthritis, meta-analysis, differentially expressed genes

## Abstract

The aim of the present study was to identify differentially expressed (DE) genes in patients with osteoarthritis (OA), and biological processes associated with changes in gene expression that occur in this disease. Using the INMEX (integrative meta-analysis of expression data) software tool, a meta-analysis of publicly available microarray Gene Expression Omnibus (GEO) datasets of OA was performed. Gene ontology (GO) enrichment analysis was performed in order to detect enriched functional attributes based on gene-associated GO terms. Three GEO datasets, containing 137 patients with OA and 52 healthy controls, were included in the meta-analysis. The analysis identified 85 genes that were consistently differentially expressed in OA (30 genes were upregulated and 55 genes were downregulated). The upregulated gene with the lowest P-value (P=5.36E-07) was S-phase kinase-associated protein 2, E3 ubiquitin protein ligase (SKP2). The downregulated gene with the lowest P-value (P=4.42E-09) was Proline rich 5 like (PRR5L). Among the 210 GO terms that were associated with the set of DE genes, the most significant two enrichments were observed in the GO categories of 'Immune response', with a P-value of 0.000129438, and 'Immune effectors process', with a P-value of 0.000288619. The current meta-analysis identified genes that were consistently DE in OA, in addition to biological pathways associated with changes in gene expression that occur during OA, which may provide insight into the molecular mechanisms underlying the pathogenesis of this disease.

## Introduction

Osteoarthritis (OA) is the most prevalent joint disease and is characterized by an abnormal remodeling of joint tissues, which is predominantly driven by inflammatory mediators within the affected joint (1,2). The pathological changes of OA primarily take place in the articular cartilage, and include cartilage degeneration, matrix degradation and synovial inflammation (3–5). Clinically, features of OA include pain, stiffness, limitation of motion, swelling and deformity (6). Synovial inflammation is hypothesized to be the primary underlying etiology in OA (3). However, the biological mechanisms associated with OA remain to be elucidated.

Microarray, a high-throughput genomics technology, has been developed in order to improve the understanding of complex interactions and networks in disease development (7). Thousands of genes on a genome-wide scale have been measured using microarray technology (8). The successful identification of gene expression signatures that may provide insights into OA pathogenesis and differentiate the diseased state from a healthy state, requires an adequate sample size and heterogeneous datasets (9). Although numerous microarray studies have generated lists of differentially expressed (DE) genes, there are inconsistencies among the results of such studies, due to the limitation of the small sample sizes involved (10).

To overcome these difficulties, meta-analysis has previously been applied to publicly-available genome-wide expression datasets from studies on a number of diseases (11–13). The use of meta-analysis may improve reliability and generalizability, and permit a more precise estimation of gene expression (11). Meta-analyses provide enhanced statistical power, thereby obtaining more robust and reliable gene signatures (7,14–17). Recently, integrative meta-analysis of expression data (INMEX), a new user-friendly microarray meta-analysis tool, has been developed to support meta-analysis of multiple gene expression datasets (18).

In order to overcome the limitations of individual studies, resolve inconsistencies in results, and reduce false-positive or false-negative associations due to random errors, a microarray meta-analysis was performed in the present study. The objective was to identify differentially expressed (DE) genes and biological processes associated with gene expression signature in OA.

## Materials and methods

### Identification of eligible gene expression datasets of OA

A search of microarray datasets was performed that examined DE genes between OA and healthy controls. The NCBI Gene Expression Omnibus (GEO) database (http://www.ncbi.nlm.nih.gov/geo/) (19) was used to identify suitable microarray datasets. The keyword 'osteoarthritis' was used for this search. Studies were included in the analysis if they: Were based on gene expression profiling of blood or synovial membrane samples; contained sufficient data to perform a meta-analysis; and included patients diagnosed with OA, based on OA classification criteria (20). The following information was extracted from each of the studies that were selected: GEO accession; sample type; platform; numbers of patients and healthy controls; and gene expression data.

### Meta-analysis of microarray datasets

All available OA microarray datasets that met the inclusion criteria were downloaded from the NCBI GEO database. Data tables containing gene expression or relative gene expression values were constructed, with genes/probes in the rows, and samples in the columns. The datasets were uploaded to INMEX (http://www.inmex.ca/INMEX) (18), and the data was subsequently annotated by converting different gene or probe ID to Entrez IDs. For each probe-set, intensity values were log-transformed and/or normalized to zero mean and unit variance, which is the normalization method for high density oligonucleotide array data, as reported by Bolstad *et al* (21). When all datasets had been uploaded, processed and annotated, a data integrity check was performed prior to the meta-analysis stage.

The random effects model presumes that different studies present substantial diversity, and evaluates between study variance as well as within study sampling error (22,23). The random effects model is used when the between-study heterogeneity is significant (23). The INMEX program was used to conduct statistical analysis (18).

### Functional analysis

The functional analysis of INMEX generates new hypotheses by exploiting characteristics of the DE gene lists identified in meta-analysis. A heat map created by 'Pattern extractor' produced gene expression profiles across different datasets/conditions.

In order to examine the functions of the genes in the gene list, gene ontology (GO) enrichment analysis was performed, which detected enriched functional attributes based on gene-associated GO terms, using the hypergeometric test (http://www.geneontology.org/) (24). Functional analysis was performed using the INMEX program (18).

## Results

### Studies included in the meta-analysis

Three GEO data sets, which met the inclusion criteria, were identified (Table I) (4,25,26). These datasets consisted of two synovial membrane datasets and one blood dataset, and included a total of 137 patients with OA and 52 controls. Selected details of the individual studies are summarized in Table I.

### Meta-analysis of gene expression in OA

**Figure 1:**
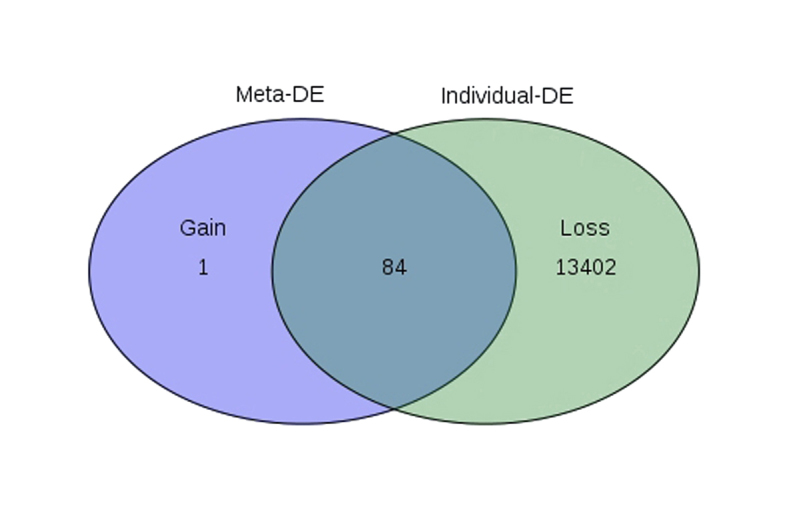
Venn diagram showing the overlap between DE genes identified from the meta-analysis (Meta-DE) and those combined from the individual data analyses (individual-DE). DE, differentially expressed.

A random effects model of effect size (ES) measures was used to integrate gene expression patterns. The present study incorporated between-study heterogeneities across studies, because the estimated Q-value was not in an approximately chi-squared distribution. DE genes with P<0.05 were selected. In the current analysis, 1 'gained' gene and 13402 'lost' genes were identified (Fig. 1). Gained genes are DE genes that were only identified in the meta-analysis (26). The single gained gene exhibited relatively weak but consistent expression profiles across the three different datasets. The large sample size obtained by consisting of the datasets made it possible declare this a DE gene with increased certainty. Lost genes are genes which were identified as DE genes in any of the individual analyses, but not in the meta-analysis. These genes also presented conflicting changes in expression profiles, or large variations across the different studies.

### Identification of differentially expressed genes in OA

A total of 85 genes were identified, which were consistently differentially expressed in OA. Among these 85 DE genes, 30 were upregulated and 55 were downregulated. A list of the top 20 upregulated and downregulated genes is shown in Table II The upregulated gene with the lowest P-value (P=5.36E-07) was S-phase kinase-associated protein 2, E3 ubiquitin protein ligase (SKP2). The downregulated gene with the lowest P-value (P=4.42E-09) was Proline rich 5 like (PRR5L).

### Identification of differentially expressed genes in the synovial membrane of patients with OA

An additional meta-analysis was performed on the results from the 2 synovial membrane samples, following exclusion of the third study, which used peripheral blood. A list of the top 20 upregulated and downregulated genes is shown in Table III. The upregulated genes with the lowest P-values (both P=0.004003) were JAZF zinc finger 1 (JAZF1) and Guanine nucleotide binding protein (G-protein), β polypeptide 4 (GNB4), which are involved in coupling membrane receptors to effector proteins, such as ion channels and enzymes (27). The downregulated gene with the largest ES (ES=2.0472; P=0.004003) was multiple inositol-polyphosphate phosphatase 1 (MINPP1). A number of the downregulated genes were related to inflammatory factors (Table III).

### Functional analysis

**Figure 2:**
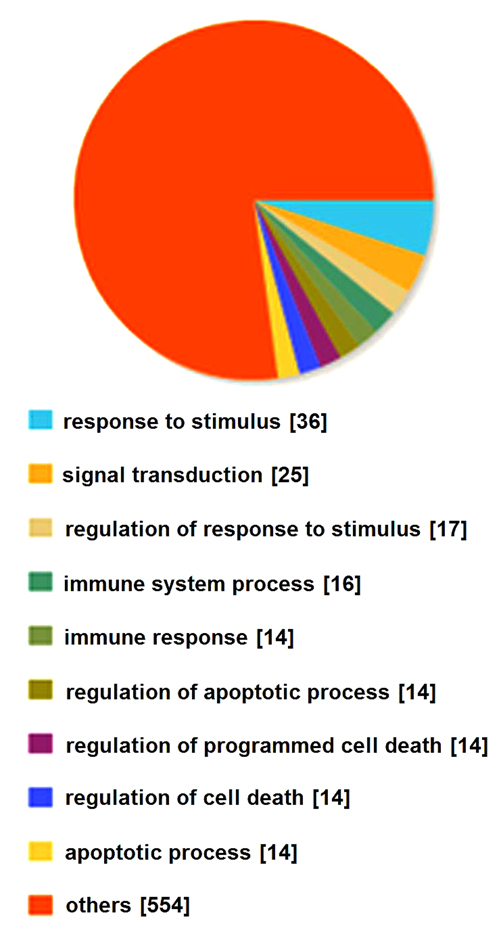
Summary of the enriched GO terms for the list of DE genes in patients with OA compared to controls. GO, gene ontology; DE, differentially expressed; OA, osteoarthritis.

GO analysis of the DE genes was performed in order to identify the biological processes associated with changes in gene expression in OA. The analysis identified 210 significant enrichments of the DE genes, which were categorized to 10 GO terms (Fig. 2). The two enrichments with the lowest P-values were in the GO category of 'Immune response', with a P-value of 0.000129438, and 'Immune effector process', with a P-value of 0.000288619. Other significant GO categories included 'Regulation of humoral immune response' (P=0.000308832), 'Regulation of immune response' (P=0.00055514) and 'Positive regulation of immune system process' (P=0.00059351; Table IV).

## Discussion

A number of genes are differentially expressed genes between patients with OA and healthy controls, and it is necessary to identify the genes that may enhance understanding of the molecular and cellular processes, which are involved in the pathogenesis of OA. Although a large quantity of data may be produced using microarray studies, the small sample size of these studies is a significant obstacle to the identification of DE genes. A meta-analysis of multiple microarray datasets increases the sample size, rendering the identification of DE genes more reliable.

In the present study, a meta-analysis was performed using three publicly available GEO datasets in order to identify common biological mechanisms involved in the pathogenesis of OA. The analysis identified 85 genes that were consistently differentially expressed in OA (30 upregulated and 55 downregulated). The upregulated gene with the largest ES was SKP2, which is known to be involved in the inhibition of cell growth and the promotion of apoptosis. Kitagawa concluded that SKP2 controls the p300–p53 signaling pathway in cancer cells (28). Furthermore, this gene encodes a member of the F-box protein family, which is characterized by a ~40 amino acid motif, the F-box (29). The downregulated gene with the lowest P-value was PRR5L, which suppresses mTOR complex 2 (mTORC2)-mediated hydrophobic motif phosphorylation of protein kinase C, but not that of protein kinase B (30). In addition, the PRR5L protein may function to modulate the activity of mTORC2 in a substrate-dependent manner (30). Actinin α 1 (ACTN1), an upregulated gene, encodes an actin-binding protein, which exerts multiple effects in a variety of cell types. ACTN1 may protect osteoclasts from tumor necrosis factor-α (TNF-α); induce apoptosis through increasing the expression of the anti-apoptotic protein, Bcl-2; activate survival signals; and promote Akt phosphorylation and NF-κB activation (31). Although it is currently unclear exactly how these genes contribute to OA, they may be useful as potential biomarkers to facilitate early diagnosis or to monitor the efficacy of treatment in this disease. A number of these genes provide insights into the molecular mechanisms underlying the pathophysiology of OA.

Although osteoarthritis (OA) is understood to be a degradative articular cartilage disease, there is increasing data demonstrating the involvement of the immune system. In recent epidemiological studies involving a large number of patients with OA, an inflammatory synovium has been shown to be involved in increased damage to the cartilage (32) and pain (33). Immune cells, such as T cells, B cells and macrophages, have been identified in the synovial tissue of patients with OA (34–36). Furthermore, immunoglobulins and immune complexes against cartilage components have been detected in the plasma, synovium and cartilage of patients with OA (37), and it has been shown that the synovium is involved in complement activation in OA (38). In the present study, 210 significantly enriched GO terms associated with the DE genes were identified using a meta-analysis. The three enriched terms with the lowest P-values were 'Immune response', 'Immune effector process' and 'Regulation of humoral immune response', which were all involved in the immune system. The identified GO terms may be grouped into a smaller number of categories: 'Response to stimulus', 'Signal transduction', 'Regulation of response to stimulus', 'Immune system process', 'Immune response', 'Regulation of apoptotic process', 'Regulation of programmed cell death', 'Regulation of cell death', 'Apoptotic process' and others. Although it is difficult to identify all the significant functional categories that are expressed differentially in OA, the GO categories identified here, merit further investigation in subsequent studies.

There were certain limitations to the present study, which ought to be considered. Firstly, heterogeneity and confounding factors may have distorted the analysis. Clinical samples may have been heterogeneous with respect to clinical activity, severity or gender. Secondly, there are differences in gene expression between tissues, such as blood and synovial membrane, that were not considered. Although an additional subgroup analysis of the synovial membrane samples was performed, this only included two studies. By contrast, the initial meta-analysis integrated the results obtained from different tissues, which should have enabled detection of the genes that may have been missed in an analysis of two studies only.

In conclusion, the meta-analysis of microarray studies that was performed in the present study, provided an overview of differential gene expression in OA; identifying 85 differential expressed genes (30 upregulated and 55 downregulated genes). Future studies to validate these genes as markers for the diagnosis and response to biological therapy for OA may provide further insight into their involvement in the development and progression of OA.

## Figures and Tables

**Table I:** Characteristics of the individual studies included in the meta-analysis.

Study (ref)	GEO accession	Patient number		Sample	Platform
		OA	Control		
1 (25)	GSE48556	106	33	Blood	Illumina HumanHT-12 V3.0 Expression Beadchip
2 (26)	GSE46750	12	12	Synovial membrane	Illumina HumanHT-12 V4.0 Expression Beadchip
3 (4)	GSE32317	19	7	Synovial membrane	Affymetrix Human Genome U133 Plus 2.0 Array

GEO, Gene Expression Omnibus; OA, osteoarthritis; ref, reference.

**Table II:** Top 20 upregulated and downregulated genes in patients with OA.

A, Top 20 upregulated genes				
Entrez ID	Gene symbol	Combined ES	P-value	Gene name
6502	SKP2	−1.1447	5.36E-07	S-phase kinase-associated protein 2, E3 ubiquitin protein ligase
23299	BICD2	−1.0632	4.24E-06	Bicaudal D homolog 2 (*Drosophila*)
8445	DYRK2	−0.8747	0.000592	Dual-specificity tyrosine-(Y)-phosphorylation regulated kinase 2
10116	FEM1B	−0.9170	0.000842	Fem-1 homolog b (*C. elegans*)
87	ACTN1	−0.8544	0.000842	Actinin, α 1
147906	DACT3	−0.8450	0.000867	Dishevelled-binding antagonist of β-catenin 3
6627	SNRPA1	−0.7981	0.002369	Small nuclear ribonucleoprotein polypeptide A'
84458	LCOR	−0.7596	0.004996	Ligand dependent nuclear receptor corepressor
55670	PEX26	−0.7396	0.007656	Peroxisomal biogenesis factor 26
284273	ZADH2	−0.7241	0.009756	Zinc binding alcohol dehydrogenase domain containing 2
2983	GUCY1B3	−0.7239	0.009756	Guanylate cyclase 1, soluble, β 3
90550	MCU	−0.7115	0.012319	Mitochondrial calcium uniporter
359845	FAM101B	−0.7042	0.014203	Family with sequence similarity 101, member B
158381	ATP8B5P	−0.7025	0.014203	ATPase, classI, type 8B, member 5, pseudogene
92014	MCART1	−0.7010	0.014203	Mitochondrial carrier triple repeat 1
57456	KIAA1143	−0.6989	0.014452	KIAA1143
51002	TPRKB	−0.6918	0.015972	TP53RK binding protein
84953	MICALCL	−0.9119	0.015993	MICAL C-terminal like
80071	CCDC15	−0.7894	0.018113	Coiled-coil domain containing 15
160	DAB2	−1.0979	0.049846	Dab, mitogen-responsive phosphoprotein, homolog 2 (*Drosophila*)

B, Top 20 downregulated genes				
Entrez ID	Gene symbol	Combined ES	P-value	Gene name
79899	PRR5L	1.3235	4.42E-09	Proline rich 5 like
5583	PRKCH	1.2343	5.08E-08	Protein kinase C, η
3683	ITGAL	1.2126	6.62E-08	Integrin, α L
5051	PAFAH2	1.0668	4.24E-06	Platelet-activating factor acetylhydrolase 2, 40kDa
24144	TFIP11	0.9820	4.31E-05	Tuftelin interacting protein 11
9595	CYTIP	0.9816	4.31E-05	Cytohesin 1 interacting protein
157567	ANKRD46	1.0119	7.59E-05	Ankyrin repeat domain 46
23294	ANKS1A	0.9547	7.60E-05	Ankyrin repeat and sterile α motif domain containing 1A
55272	IMP3	1.2776	0.001032	IMP3, U3 small nucleolar ribonucleoprotein
80213	TM2D3	1.0182	0.001425	TM2 domain containing 3
474344	GIMAP6	1.1551	0.004506	GTPase, IMAP family member 6
55303	GIMAP4	1.2787	0.008432	GTPase, IMAP family member 4
10866	HCP5	1.1647	0.001232	HLA complex P5 (non-protein coding)
54499	TMCO1	1.0355	0.014452	Transmembrane and coiled-coil domains 1
951	CD37	1.0423	0.023921	CD37 molecule
50650	ARHGEF3	1.0568	0.025483	ρ guanine nucleotide exchange factor (GEF) 3
56833	SLAMF8	1.0983	0.033534	SLAM family member 8
65992	DDRGK1	1.4283	0.033711	DDRGK domain containing 1
112858	TP53RK	1.7509	0.036334	TP53 regulating kinase
4236	MFAP1	1.2892	0.047164	Microfibrillar-associated protein 1

ES, effect size; OA, osteoarthritis.

**Table III:** Top 10 upregulated and downregulated genes in the synovial membrane of patients with OA.

A, Upregulated genes				
Entrez ID	Gene symbol	Combined ES	P-value	Gene name
221895	JAZF1	−1.6865	0.004003	JAZF zinc finger 1
59345	GNB4	−1.6774	0.004003	Guanine nucleotide binding protein (G protein), β polypeptide 4
3070	HELLS	−1.6193	0.005903	Helicase, lymphoid-specific
9749	PHACTR2	−1.5892	0.005903	Phosphatase and actin regulator 2
9645	MICAL2	−1.5726	0.006072	Microtubule associated monooxygenase, calponin and LIM domain containing 2
10974	C10orf116	−1.5715	0.006072	Adipogenesis regulatory factor
283310	OTOGL	−1.5624	0.006072	Otogelin-like
26230	TIAM2	−1.5531	0.006563	T-cell lymphoma invasion and metastasis 2
51194	IPO11	−1.5378	0.006822	Importin 11
1404	HAPLN1	−1.5176	0.007348	Hyaluronan and proteoglycan link protein 1

B, Downregulated genes				
Entrez ID	Gene symbol	Combined ES	P-value	Gene name
27242	TNFRF21	1.8378	0.003981	Tumor necrosis factor receptor superfamily, member 21
84225	ZMYND15	1.8026	0.003981	Zinc finger, MYND-type containing 15
9562	MINPP1	2.0472	0.004003	Multiple inositol-polyphosphate phosphatase 1
3600	IL15	1.9554	0.004003	Interleukin 15
3487	IGFBP4	1.7005	0.004003	Insulin-like growth factor binding protein 4
54504	CPVL	1.6845	0.004003	Carboxypeptidase, vitellogenic-like
9450	LY86	1.6796	0.004003	Lymphocyte antigen 86
147798	TMC4	1.6305	0.004882	Transmembrane channel-like 4
834	CASP1	1.5939	0.005903	Caspase 1, apoptosis-related cysteine peptidase
83401	ELOVL3	1.8314	0.006822	ELOVL fatty acid elongase 3

ES, effect size; OA, osteoarthritis.

**Table IV:** Top 10 enriched GO terms among the DE genes in patients with OA compared with controls.

GO ID	Term	P-value	Genes
GO:0006955	Immune response	0.000129438	ITGAL, GIMAP5, INPP5D, LAX1, GZMA, IRF1, CST7, SKAP1, CD55, TNFRSF4, CD37, CX3CR1, HLA-C, CXCL3
GO:0002252	Immune effector process	0.000288619	ITGAL, GIMAP5, INPP5D, IRF1, CD55, TNFRSF4, CD37, CX3CR1
GO:0002920	Regulation of humoral immune response	0.000308832	GIMAP5, CD55, CD37
GO:0042981	Regulation of apoptotic process	0.000510947	PRKCH, SKP2, PAFAH2, TMBIM6, GIMAP5; DYRK2, ACTN1, FEM1B, INPP5D, GZMA, IRF1, TNFRSF4, CX3CR1, ARHGEF3
GO:0051250	Negative regulation of lymphocyte activation	0.000517654	GIMAP5, INPP5D, LAX1, IRF1
GO:0045589	Regulation of regulatory T cell differentiation	0.000531439	GIMAP5, IRF1
GO:0043067	Regulation of programmed cell death	0.000551712	PRKCH, SKP2, PAFAH2, TMBIM6, GIMAP5, DYRK2, ACTN1, FEM1B, INPP5D, GZMA, IRF1, TNFRSF4, CX3CR1, ARHGEF3
GO:0050776	Regulation of immune response	0.000555143	PRKCH, ITGAL, GIMAP5, INPP5D, IRF1, SKAP1, CD55, CD37, HLA-C
GO:0002684	Positive regulation of immune system process	0.000593511	PRKCH, ITGAL, GIMAP5, INPP5D, IRF1, SKAP1, CD55, TNFRSF4, CD37
GO:0010941	Regulation of cell death	0.000708372	PRKCH, SKP2, PAFAH2, TMBIM6, GIMAP5, DYRK2, ACTN1, FEM1B, INPP5D, GZMA, IRF1, TNFRSF4, CX3CR1, ARHGEF3

GO, gene ontology; DE, differentially expressed; OA, osteoarthritis; ITGAL, Integrin, α L; GIMAP5, GTPase, IMAP family member 5; INPP5D, inositol polyphosphate-5-phosphatase; LAX1, lymphocyte transmembrane adaptor 1; GZMA, granzyme A; IRF1, interferon regulatory factor 1; SKAP1, src kinase associated phosphoprotein 1; TNFRSF4, tumor necrosis receptor superfamily, member 4; CX3CR1, CX3C chemokine receptor 1; HLA-C, humna leukocyte antigen C; CXCL3, chemokine (C-X-C motif) ligand 3; PRKCH, protein kinase C, η; SKP2, S-phase kinase-associated protein 2, E3 ubiquitin protein ligase; PAFAH2, platelet-activating factor acetylhydrolase 2, 40kDa; TMBIM6, transmembrane BAX inhibitor motif containing 6; DYRK2, Dual-specificity tyrosine-(Y)-phosphorylation regulated kinase 2; ACTN1, Actinin, α 1; FEM1B, Fem-1 homolog b (*C. elegans*); ARHGEF3, ρ guanine nucleotide exchange factor (GEF) 3.
